# Predicting Risk of Hypoglycemia in Patients With Type 2 Diabetes by Electronic Health Record–Based Machine Learning: Development and Validation

**DOI:** 10.2196/36958

**Published:** 2022-06-16

**Authors:** Hao Yang, Jiaxi Li, Siru Liu, Xiaoling Yang, Jialin Liu

**Affiliations:** 1 Information Center West China Hospital Sichuan University Chengdu China; 2 Department of Clinical Laboratory Medicine Jinniu Maternity and Child Health Hospital of Chengdu Chengdu China; 3 Department of Biomedical Informatics Vanderbilt University Medical Center Nashville, TN United States; 4 West China School of Nursing, Endocrinology and Metabolism Department West China Hospital Sichuan University Chengdu China; 5 Department of Medical Informatics West China Medical School Chengdu China

**Keywords:** diabetes, type 2 diabetes, hypoglycemia, learning, machine learning model, EHR, electronic health record, XGBoost, natural language processing

## Abstract

**Background:**

Hypoglycemia is a common adverse event in the treatment of diabetes. To efficiently cope with hypoglycemia, effective hypoglycemia prediction models need to be developed.

**Objective:**

The aim of this study was to develop and validate machine learning models to predict the risk of hypoglycemia in adult patients with type 2 diabetes.

**Methods:**

We used the electronic health records of all adult patients with type 2 diabetes admitted to West China Hospital between November 2019 and December 2021. The prediction model was developed based on XGBoost and natural language processing. F1 score, area under the receiver operating characteristic curve (AUC), and decision curve analysis (DCA) were used as the main criteria to evaluate model performance.

**Results:**

We included 29,843 patients with type 2 diabetes, of whom 2804 patients (9.4%) developed hypoglycemia. In this study, the embedding machine learning model (XGBoost3) showed the best performance among all the models. The AUC and the accuracy of XGBoost are 0.82 and 0.93, respectively. The XGboost3 was also superior to other models in DCA.

**Conclusions:**

The Paragraph Vector–Distributed Memory model can effectively extract features and improve the performance of the XGBoost model, which can then effectively predict hypoglycemia in patients with type 2 diabetes.

## Introduction

Diabetes is a serious long-term disease. The global prevalence of diabetes in people aged 20-79 years is estimated to be 10.5% (536.6 million) in 2021 and will rise to 12.2% (783.2 million) by 2045. Global health expenditures related to diabetes are estimated US $966 billion in 2021 and projected to reach US $1054 billion by 2045 [1]. Diabetes continues to be a major clinical and public health concern [2].

Hypoglycemia (blood glucose<3.9 mmol/L or 70 mg/dL) is a common adverse event of diabetes treatment. Hospital hypoglycemia occurs in 3%-18% of hospitalized diabetic patients [3]. Severe hypoglycemia usually causes potentially life-threatening complications and is associated with an increase length of stay and mortality [4,5]. Hypoglycemia is especially common in older patients with diabetes [5], and the risk doubles every decade after the age of 60 years [6]. Many factors can lead to a high risk of hypoglycemia in older patients, including physiological changes in drug metabolism, age-related decline in renal function, cognitive decline, an increase in comorbidity, and potential overtreatment [7,8]. Since there are many risk factors that induce hypoglycemia in patients with diabetes, and some risk factors may also change during hospitalization, it is a challenge to identify and prevent hypoglycemia in people with diabetes [9,10].

In recent years, machine learning has been widely used for hypoglycemia prediction. For example, Schroeder et al [11] employed the Cox prediction model for the 6-month risk of hypoglycemia. Karter et al [12] developed a tool to identify patients with type 2 diabetes at a high risk of hypoglycemia. Plis et al [13] described a support vector regression model for predicting hypoglycemic events. Furthermore, Jin et al [14] have integrated deep learning with natural language processing (NLP) to automatically detect hypoglycemic events from electronic health record (EHR) notes.

Although numerous hypoglycemia prediction models have been developed, there is still a need to improve the accuracy and effectiveness of hypoglycemia prediction. In this study, we developed XGBoost ensembling NLP to predict the risk of hypoglycemia in hospitalized patients with type 2 diabetics, using data readily available in the EHRs.

## Methods

Our cohort included patients with type 2 diabetes from West China Hospital of Sichuan University. All patient data were obtained from the hospital’s EHR system.

### Ethics Approval

The study was approved by the Medical Ethics Committee of West China Hospital Sichuan University (2020-608). West China Hospital is a large teaching hospital with 4300 beds and a leading medical center of western China [15].

### Patients

**Figure 1:**
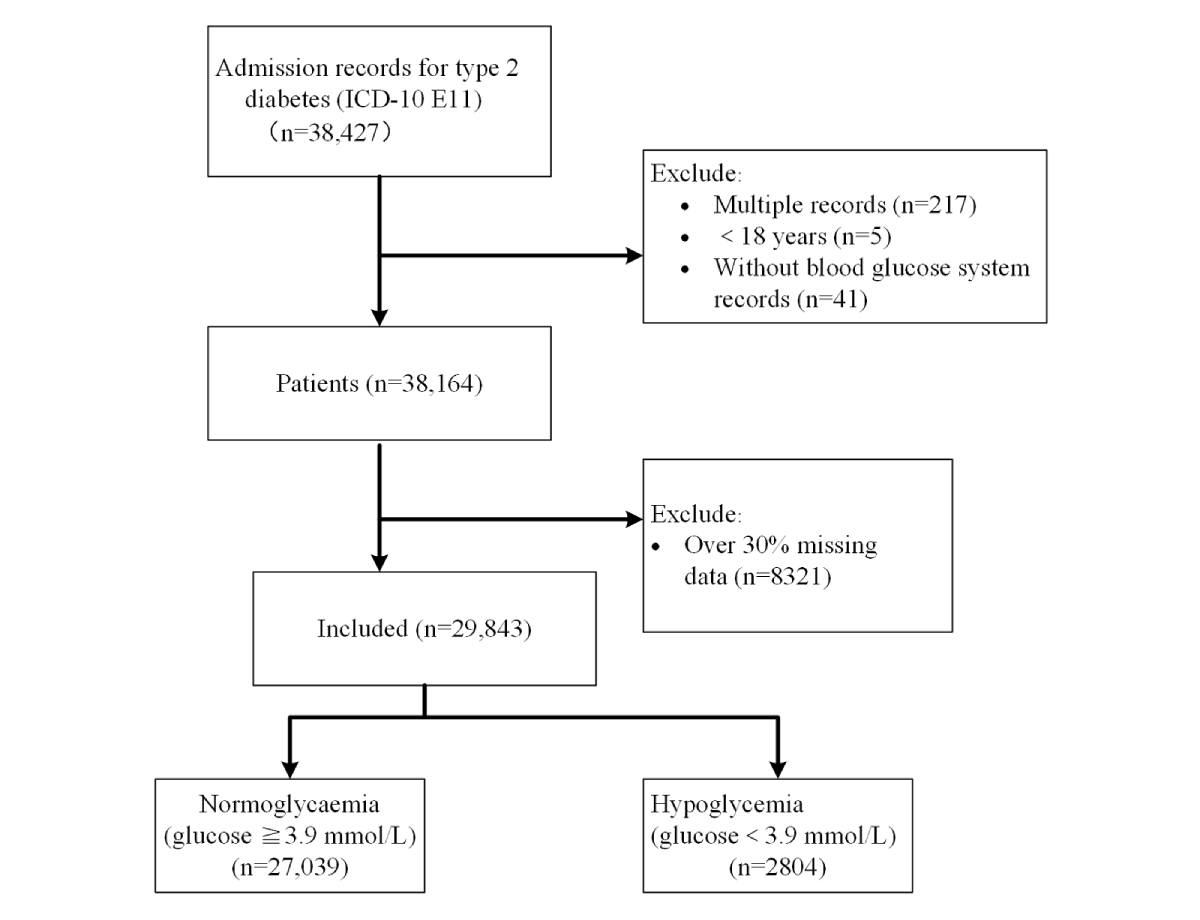
The patient selection process.

We performed a retrospective analysis of the available EHR of all patients with type 2 diabetes who were admitted to West China Hospital between November 2019 and December 2021. With the protection of patient privacy, only data related to the patient’s hospitalization were retrieved, and the diagnosis was established based on International Classification of Diseases, 10th revision (ICD-10). The following inclusion criteria were used: (1) all patients with type 2 diabetes based on ICD-10, E11 (type 2 diabetes mellitus) with a hospital stay>24 hours; (2) patients aged 18 years or older. Patients with more than 30% missing values were excluded from the analysis [16]. The patient selection process is shown in Figure 1.

### Variables Analyzed

The variables used to predict the risk of hypoglycemia in patients with type 2 diabetes included various demographic, laboratory, and clinical variables, as well as EHR notes. The extraction of variables was based on experts’ opinion and our research [16-20]. These variables were collected during the first 24 hours of admission. Through data preprocessing, we analyzed some missing values (Table 1). Random forest regression was used to handle all missing numerical variables.

**Table 1:** Statistics of missing values (N=29,843).

Features	Missing data, n (%)
Red blood cell count	1860 (6.2)
Hemoglobin	1858 (6.2)
Blood platelet count	1883 (6.3)
White blood cell count	1858 (6.2)
Total protein	1791 (6.0)
Albumin	1768 (5.9)
Globulin	1812 (6.1)
Urea	1755 (5.9)
Alanine aminotransferase	1821 (6.1)
Aspartate aminotransferase	1809 (6.1)
Cholesterol	2126 (7.1)
High-density lipoprotein	2128 (7.1)
Low-density lipoprotein	2131 (7.1)
Sodium	1516 (5.1)
Chlorine	1585 (5.3)
Thrombin time	3970 (13.3)
Creatinine	1749 (5.9)
Uric acid	1769 (5.9)
C-reactive protein	18,249 (61.1)
Procalcitonin	20,101 (67.3)
Glycosylated hemoglobin or HbA_1c_^a^	14,410 (48.3)
Prothrombin time	3725 (12.5)
Activated partial thromboplastin time	3779 (12.7)

^a^HbA_1c_: glycated hemoglobin.

### Variable Selection

**Figure 2:**
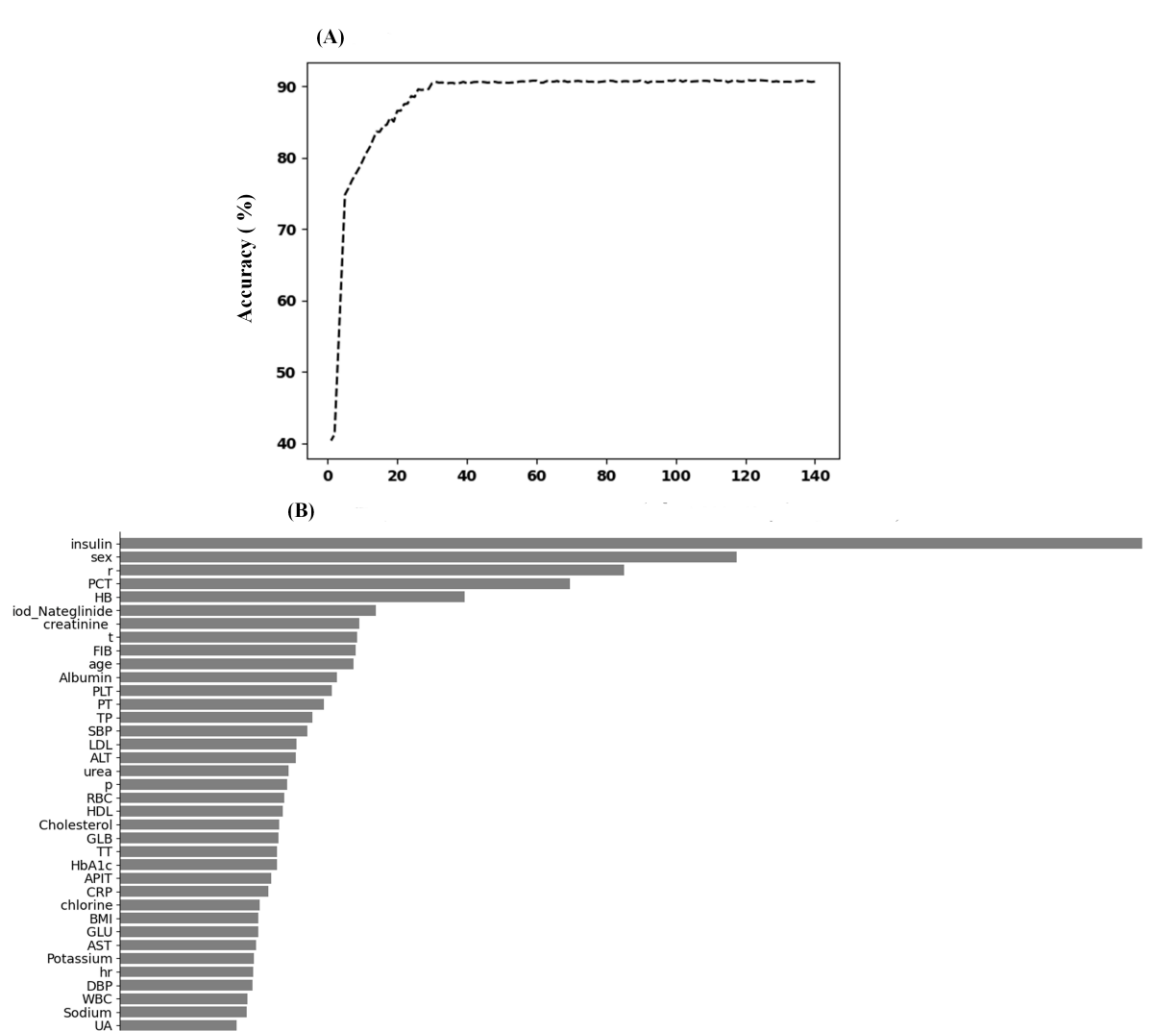
The weights of variables importance. ALT: alanine aminotransferase; APIT: activated partial thromboplastin time; AST: aspartate aminotransferase; CRP: C-reactive protein; DBP: diastolic blood pressure; FIB: fibrinogen; GLB: globulin; GLU: glucose; HB: hemoglobin; HbA_1c_: glycated hemoglobin; HDL: high-density lipoprotein; hr: heart rate; iod-Nateglinide: iodine urea and Nateglinide; LDL: low-density lipoprotein; p: pulse; PCT: procalcitonin; PLT: blood platelet count; PT: prothrombin time; r: respiratory rate; RBC: red blood cell count; SBP: systolic pressure; t: body temperature; TP: total protein; TT: thrombin time; UA: uric acid; WBC: white blood cell count. (A) the curve between the number of features and accuracy. (B) the weights of variables importance (when accuracy is up to 90%).

After extracting all the variables, the parameter of feature importance in XGBoost was used to select and filter important variables [21]. The parameters were set as follows: the number of estimators was 100 and max depth was set to 6. Ultimately, 37 predictive variables and their weights were selected from 176 variables (Figure 2).

### Data Imbalance

To overcome the data imbalance between the group with hypoglycemia and the normoglycemic control group, we used the Adaptive Synthetic (ADASYN) sampling method [22] to oversample the group with hypoglycemia to generate a portion of data that was comparable to the data from the normoglycemic group. The method for imbalanced learning was used to generate a low sample size to improve class imbalance. We used 5-fold cross-validation and sample balancing with ADASYN for each stratified training set. ADASYN was implemented using Imblearn in Python (Version 0.9.0; imbalanced-learn documentation) [23]. The sampling ratio was set to 1.

### Embedding Models

**Figure 3:**
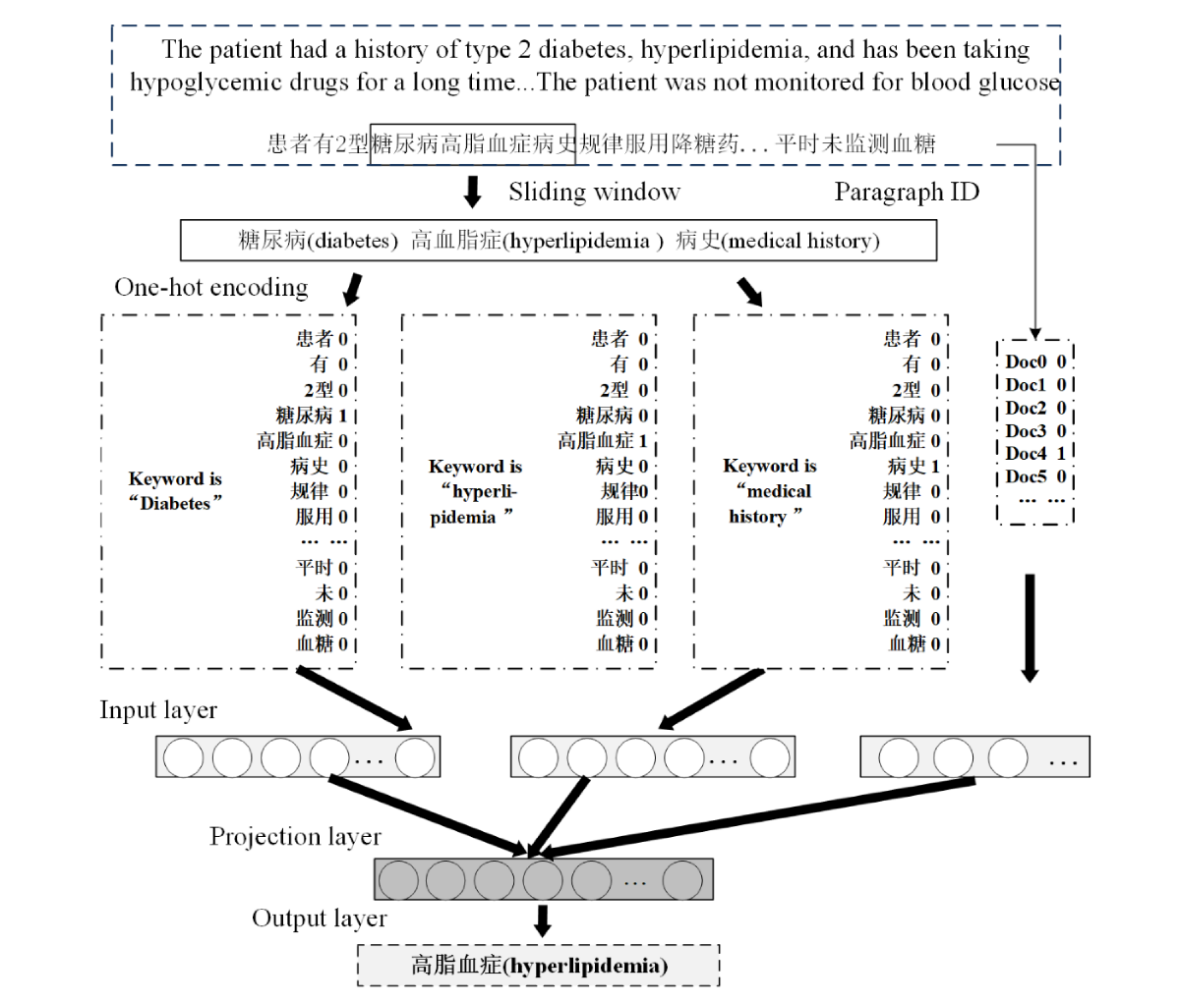
Training process of Paragraph Vector–Distributed Memory (PV-DM) model.

We used a Python implementation of the Paragraph Vector model available at Gensim [24] and trained 100-dimensional vectors on our corpus. Due to the large computing time of training these corpora and because they are unlabeled corpora, we trained the distributed memory model of paragraph vectors or Paragraph Vector–Distributed Memory (PV-DM) [25] based on textual data from patients with diabetes (Figure 3).

The doc2vec model was used to train and feature map the chief complaints (CCs), history of present illness (HPI), and family history (FH) in the EHR. The results were feature fused into XGBoost model [21] to generate XGBoost1 (XGBoost+CC), XGBoost2 (XGBoost+CC+HPI), and XGBoost3 (XGBoost+CC+HPI+FH).

### Statistical Analysis

As clinical indicators, categorical variables were shown as counts and percentages, and continuous variables were shown as means and SDs. Comparisons between groups were analyzed by a 2-tailed *t* test for continuous variables and chi-square test for categorical variables. All statistical analyses were carried out in R software (version 4.1.2; R Core Team). The statistical significance was considered as *P*<.05. The computing environment of this study includes central processing unit i7-7800x; memory 16 GB; operating system Windows 11, build 22598.200; and Python programming language.

## Results

### Participant Characteristics

The cohort included 29,843 patients with type 2 diabetes, of whom 2804 (9.4%) patients developed hypoglycemia. Among the 29,843 patients, the proportion of female patients in the group with hypoglycemia (n=1065, 38.0%) was higher than that in the normoglycemia group (n=9479, 35.1%; *P*=.002). The BMI of patients in the hypoglycemia and normoglycemia groups were 23.6 (SD 5.24) and 24.3 (SD 4.26), respectively. Statistically, the BMI of patients in the normoglycemia group was significantly higher than that of patients in the hypoglycemia group (*P*<.001). The proportion of insulin use in patients in hypoglycemia group (n=1575, 56.2%) was much higher than that for patients in the normoglycemia group (n=7306, 27.0%). In addition, the proportion of patients taking sulfonylureas or Nateglinide in the hypoglycemia group (n=1382, 49.3%) was also higher than that in the normoglycemia group (n=9273, 34.3%), which was a statistically significant difference (*P*<.001). The demographics of patients in normoglycemia and hypoglycemia groups are shown in Table 2.

**Table 2:** Demographics of patients with diabetes (N=29,843).

Variables		Normoglycemia (blood glucose>3.9 mmol/L; n=27,039)	Hypoglycemia (blood glucose<3.9 mmol/L; n=2804)	*P* values	
**Sex, n (%)**					.002
	Female	9479 (35.1)	1065 (38)		
	Male	17,560 (64.9)	1739 (62)		
Age (years), mean (SD; range)		64.2 (12.3; 18-104)	64.8 (12.6; 19-98)	.03	
BMI, mean (SD)		24.3 (4.26)	23.6 (5.24)	<.001	
**Insulin, n (%)**					<.001
	No	19,733 (73)	1229 (43.8)		
	Yes	7306 (27)	1575 (56.2)		
**Sulfonylureas or Nateglinide, n (%)**					<.001
	No	17,766 (65.7)	1422 (50.7)		
	Yes	9273 (34.3)	1382 (49.3)		

### Feature Selection

**Figure 4:**
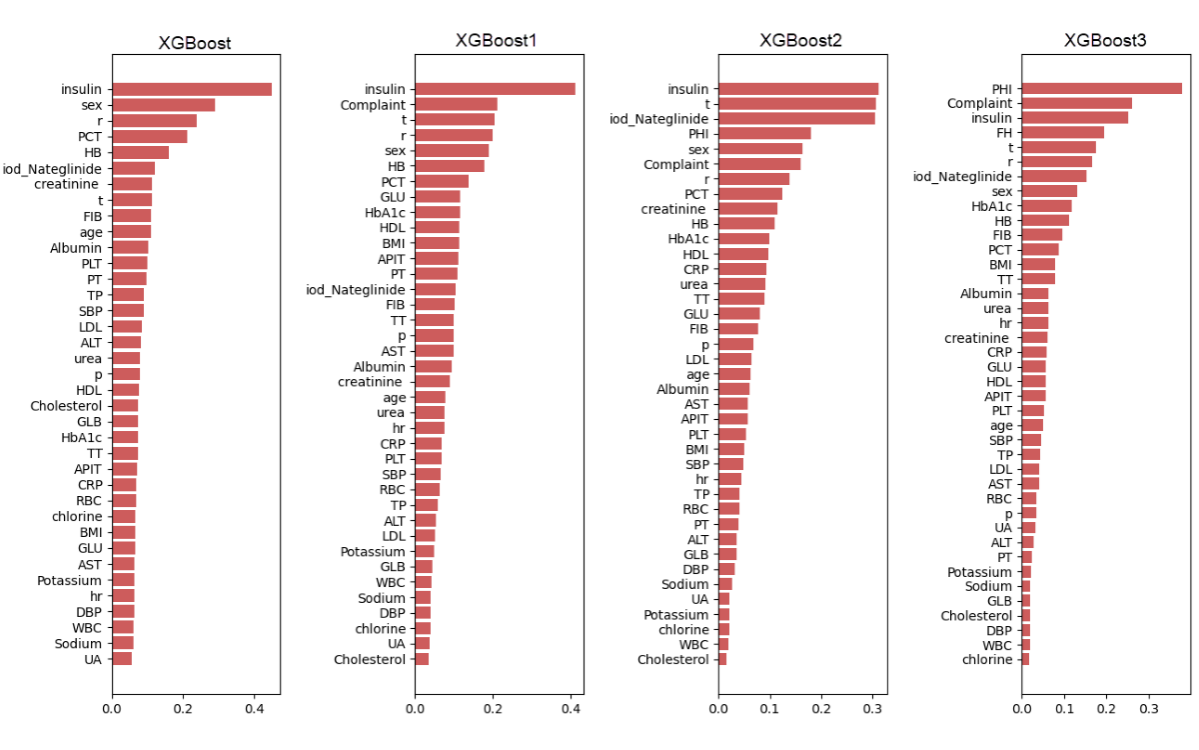
Weight of the variables in the different models. ALT: alanine aminotransferase; APIT: activated partial thromboplastin time; AST: aspartate aminotransferase; CRP: C-reactive protein; DBP: diastolic blood pressure; FIB: fibrinogen; GLB: globulin; GLU: glucose; HB: hemoglobin; HDL: high-density lipoprotein; hr: heart rate; iod-Nateglinide: Iodine urea and Nateglinide; LDL: low-density lipoprotein; p: pulse; PCT: procalcitonin; PLT: blood platelet count; PT: prothrombin time; r: respiratory rate; RBC: red blood cell count; SBP: systolic pressure; t: body temperature; TP: total protein; TT: thrombin time; UA: uric acid; WBC: white blood cell count.

We applied XGBoost and its ensemble models for feature selection to discard noninformative features and retain important features (Figure 4). Finally, 37 features were selected from 176 features. In the XGBoost model, insulin was the most important predictor variable among all the predictor variables, followed by sex, respiratory rate, Procalcitonin, and hemoglobin (Figure 4). However, these variables had different weights in XGBoost1, XGBoost2, and XGBoost3 (Figure 4).

### Model Performance

**Figure 5:**
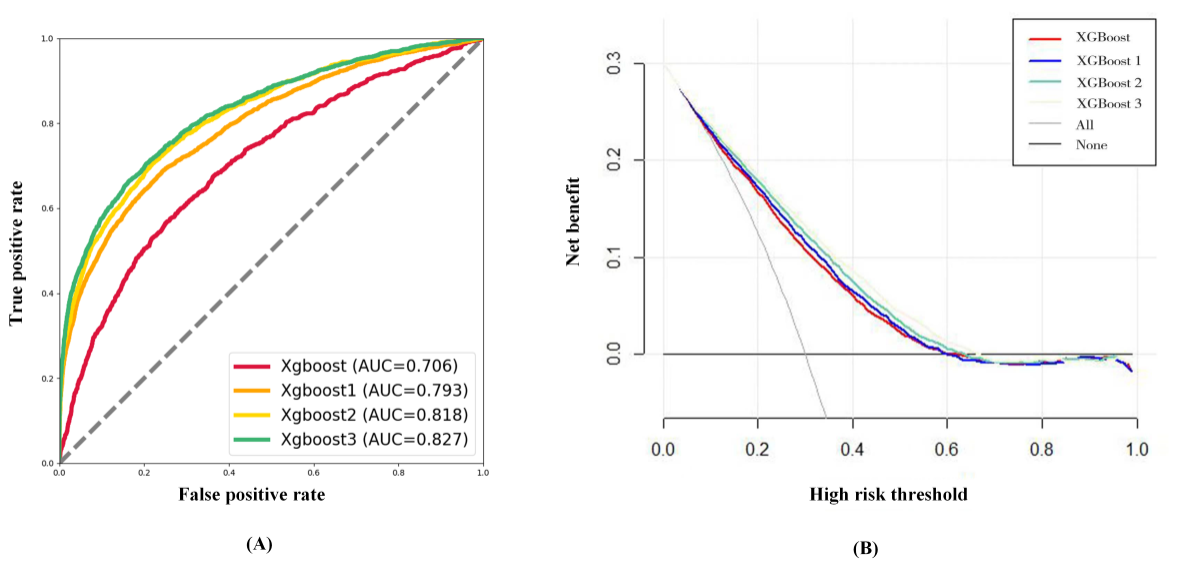
Comparison between the change detection algorithm (CDA) and receiver operating characteristic (ROC) curve of different models. (A) The ROC curve of the 4 models. (B) The DCA curve of the 4 models.

Table 3 shows the results of the 4 machine learning methods after 5-fold cross-validation. The area under the receiver operating characteristic curve (AUC=0.822) and accuracy (0.934) of the XGBoost3 were higher than all other models. The XGboost3 was superior to other models in terms of model performance, which was evaluated using AUC and decision curve analysis [26] (Figure 5).

**Figure 6:**
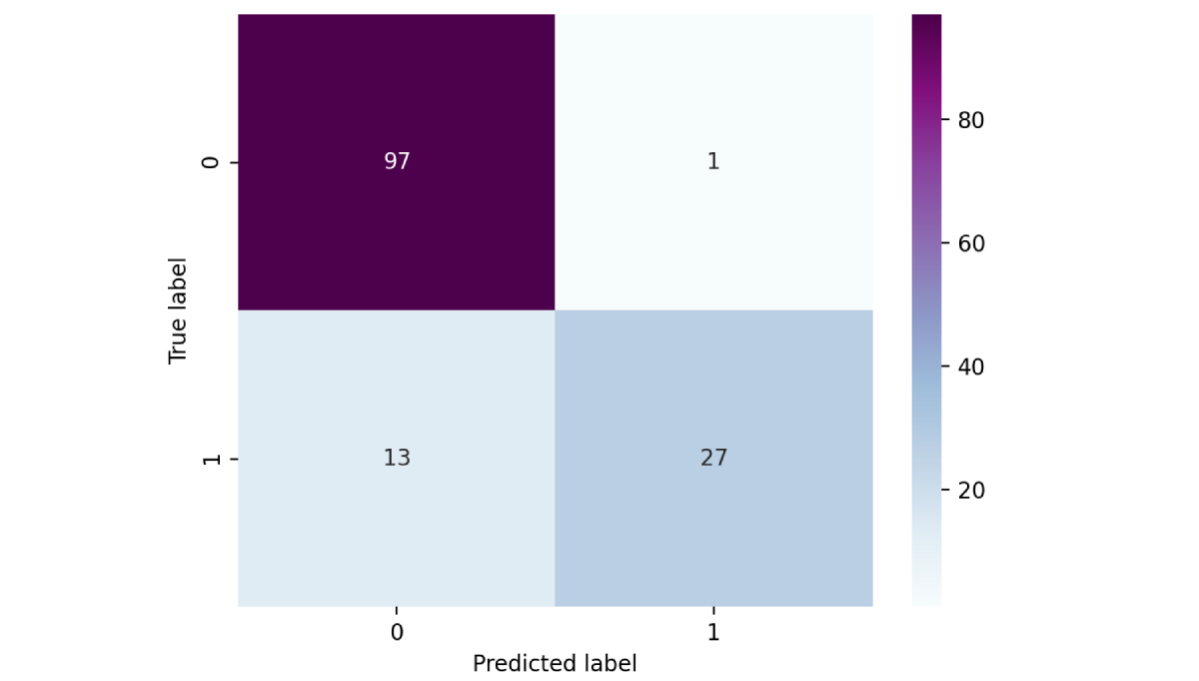
The confusion matrix of XGBoost3.

The oversample may have an impact on the accuracy of the test set. After completing the model training, we sampled 138 adult patients with type 2 diabetes (hypoglycemia=28, nonhypoglycemia=110) in West China Hospital from January to March 2022 for validation. The results showed that the prediction accuracy rate reaches 89.86%. The confusion matrix is shown in Figure 6.

**Table 3:** Accuracy and area under the receiver operating characteristic curve (AUC) of different models.

Model	Embedding method	AUC, mean (SD)	Accuracy, mean (SD)	*P* value
XGBoost	XGBoost	0.718 (0.0014)	0.892 (0.002)	N/A^a^
XGBoost1	XGBoost+CC^b^	0.785 (0.0012)	0.919 (0.002)	<.001 vs XGBoost
XGBoost2	XGBoost+CC+HPI^c^	0.817 (0.0023)	0.928 (0.001)	<.001 vs XGBoost <.001 vs XGBoost1
XGBoost3	XGBoost+CC+HPI+FH^d^	0.822 (0.0024)	0.934 (0.002)	<.001 vs XGBoost <.001 vs XGBoost1 <.001 vs XGBoost2

^a^N/A: not applicable.

^b^CC: chief complaints.

^c^HPI: history of present illness.

^d^FH: family history.

## Discussion

### Principal Findings

In this study, we used only some common types of features within 24 hours of patient admission to develop a hypoglycemia prediction model, because the earlier hypoglycemia is predicted or detected, the better we can avoid it. This study found that in women, patients at an older age, patients with low BMI, and those using insulin or various hypoglycemic drugs, the risk of hypoglycemia developing in type 2 diabetes increases. There was a statistical difference (*P*<.001). Some studies have shown that these factors increase significantly in the incidence of hypoglycemia in patients with type 2 diabetes [27-29]. This may be related to the higher risk of sulfonylurea-related hypoglycemia in women compared to men [30]. One possible reason for this is the pharmacokinetics and pharmacodynamics of sulfonylureas in women [31]. In patients with type 2 diabetes, low BMI may be associated with reduced insulin resistance [32]. Patients with obesity can benefit from the same type of antidiabetic drugs that patients with low or normal weight use [33]. This phenomenon is known as the “obesity paradox,” but the mechanism is unknown [34]. This indicates that a standard BMI or overweight are key determinants in reducing the risk of severe hypoglycemia in patients with type 2 diabetes [35].

We developed a hypoglycemia risk prediction model based on XGBoost integrated PV-DM, which can be applied to patients with type 2 diabetes. The result showed that XGBoost3 has the largest AUC and highest accuracy to predict hypoglycemia. There is a significant difference between this model and other models (*P*<.001). Consistent with previous research [36], combining numerical variables with textual data from EHR can effectively improve the predictive performance of the model. Applying this model to clinical practice could help physicians adjust hypoglycemic drugs based on patient characteristics and hypoglycemia risk factors. This study demonstrates that the inclusion of EHR increases the prognostic accuracy of hypoglycemia in patients with diabetes, providing a more comprehensive and optimized method for predicting hypoglycemic events.

This study also has some limitations. First, the study was carried out in a single institution, and the performance of the model and the distribution of covariates may differ when applied to a sample from a different institution. Second, this study involved Chinese patients. Due to ethnic differences, the results of this study need to be further verified in other ethnic groups.

### Conclusions

We developed a multivariate risk prediction model to predict the occurrence of hypoglycemia in patients with type 2 diabetes. In this prediction model, the PV-DM model can effectively extract the EHR notes and improve the performance of the XGBoost model.

The predictive model can help predict the occurrence of hypoglycemia in patients with type 2 diabetes and provide clinicians with an effective way to prevent hypoglycemia in patients with diabetes. In future research, we will focus on external validation of this model in a larger cohort of patients with type 2 diabetes and explore combining state-of-the-art methods in NLP with deep learning to enhance the model’s predictive power.

## Abbreviations

ADASYN: adaptive synthetic
AUC: area under the receiver operating characteristic curve
CC: chief complaints
DCA: decision curve analysis
EHR: electronic health record
FH: family history
HPI: history of present illness
ICD-10: International Classification of Diseases, 10th revision
NLP: natural language processing
PV-DM: Paragraph Vector–Distributed Memory
